# Angiogenic Effects and Crosstalk of Adipose-Derived Mesenchymal Stem/Stromal Cells and Their Extracellular Vesicles with Endothelial Cells

**DOI:** 10.3390/ijms221910890

**Published:** 2021-10-08

**Authors:** Swarna Rautiainen, Timo Laaksonen, Raili Koivuniemi

**Affiliations:** Drug Research Program, Division of Pharmaceutical Biosciences, Faculty of Pharmacy, University of Helsinki, Viikinkaari 5E, 00790 Helsinki, Finland; swarnahelena@gmail.com (S.R.); timo.laaksonen@helsinki.fi (T.L.)

**Keywords:** adipose-derived mesenchymal stem/stromal cell, endothelial cell, co-culture, cell transplantation, co-transplantation, extracellular vesicle, angiogenesis, ischemia, wound healing

## Abstract

Adipose-derived mesenchymal stem/stromal cells (ASCs) are an adult stem cell population able to self-renew and differentiate into numerous cell lineages. ASCs provide a promising future for therapeutic angiogenesis due to their ability to promote blood vessel formation. Specifically, their ability to differentiate into endothelial cells (ECs) and pericyte-like cells and to secrete angiogenesis-promoting growth factors and extracellular vesicles (EVs) makes them an ideal option in cell therapy and in regenerative medicine in conditions including tissue ischemia. In recent angiogenesis research, ASCs have often been co-cultured with an endothelial cell (EC) type in order to form mature vessel-like networks in specific culture conditions. In this review, we introduce co-culture systems and co-transplantation studies between ASCs and ECs. In co-cultures, the cells communicate via direct cell–cell contact or via paracrine signaling. Most often, ASCs are found in the perivascular niche lining the vessels, where they stabilize the vascular structures and express common pericyte surface proteins. In co-cultures, ASCs modulate endothelial cells and induce angiogenesis by promoting tube formation, partly via secretion of EVs. In vivo co-transplantation of ASCs and ECs showed improved formation of functional vessels over a single cell type transplantation. Adipose tissue as a cell source for both mesenchymal stem cells and ECs for co-transplantation serves as a prominent option for therapeutic angiogenesis and blood perfusion in vivo.

## 1. Introduction

Endothelial dysfunction is known to be a common denominator of various pathophysiological conditions such as diabetes mellitus, coronary heart disease and stroke [1,2,3]. Endothelial dysfunction results in ischemia in the nearby tissues, thus impairing cellular regeneration mechanisms, which can eventually lead to tissue necrosis. Adipose-derived mesenchymal stem/stromal cells (ASCs) provide a promising option to treat endothelial dysfunction since they can promote angiogenesis through growth factor and extracellular vesicle production and differentiate into either endothelial cells or pericyte-like cells [4,5,6]. Due to these favorable characteristics, and because they are easy to harvest, ASCs are a potential option for cell therapies.

Therapeutic angiogenesis serves as a promising approach in the treatment of ischemic diseases. Traditionally, therapeutic angiogenesis has been cytokine-based, but the failure to provide efficient therapy methods has proven to be a major challenge [7]. In recent years, therapeutic angiogenesis has shifted towards stem cell transplantation. Current research aims to develop methods that can aid the tissue regeneration process in ischemic conditions via the administration of healthy, pro-angiogenic stem cells or their extracellular vesicles either directly or in a vascular graft. The administration is commonly preceded by seeding of the cells on a synthetic or biomaterial scaffold and expansion of the cells in vitro, which could augment the bioactivity and angiogenic capacity of cells. Furthermore, the scaffold or prevascular structures in the graft could provide better cell survival after transplantation, which is a major obstacle in cell therapies.

Different co-culture systems between ASCs and endothelial cells have been applied in regenerative medicine and tissue engineering in order to discover the environmental and biochemical mechanisms and angiogenic factors needed to establish mature and stable capillary network growth [8,9,10,11]. Regardless of the environmental cues, it has been discovered that ASCs often promote endothelial cell tube formation in co-culture systems. However, challenges remain regarding how to form stable tubular structures for in vivo delivery purposes.

Extracellular vesicles (EVs) released by different cell types facilitate cell-to-cell communication and are important mediators in both normal physiology and in pathophysiological conditions. Recent research has established EV-mediated transport of microRNAs (miRNA, miR), mRNAs and proteins to be an important part of the angiogenic process [12,13]. In particular, ASC-derived EVs have been found to have a cargo that promotes angiogenesis, thus making ASC EVs extremely interesting for cell-free therapies, which could avoid the disadvantages of current cell transplantation procedures.

This review aims to discuss what is currently known about ASCs’ ability to promote angiogenesis through paracrine secretion and differentiation into endothelial cells or pericytes and the interactions between ASCs and endothelial cells in the angiogenesis-promoting process in cell co-cultures and with respect to their combined transplantation in vivo. As a novel possibility for therapeutic purposes, ASC-derived extracellular vesicles and their angiogenesis-promoting qualities and cellular mechanisms will be discussed.

## 2. Endothelial Cells

The purpose of a vascular network is to ensure proper delivery of oxygen and nutrients to all tissues of the body and provide a means to dispose of metabolic waste [14]. In addition, it has a crucial role in the function of the immune system and metabolic processes, delivering cells of the immune system and hormones to their target location. Endothelial cells (ECs) have an essential role in vascular homeostasis, regulation of blood flow and immune cell filtration [15,16,17]. Several factors secreted by endothelial cells, such as nitric oxide, endothelin and prostacyclin, are responsible for the maintenance of vascular homeostasis [18]. These factors have a key role in regulating vascular tone through smooth muscle contraction and relaxation, platelet activity and other coagulation-related factors [18]. In addition, endothelial cells facilitate angiogenesis, the sprouting of new blood vessels from already existing vessels, which is of crucial importance when aiming to restore the proper function of tissue after damage [15].

The endothelium may become damaged due to physical, biological or biochemical injuries [1]. Injuries to the endothelium are characterized by ischemia resulting from a shortage of arterial blood supply to the tissue [7]. Therefore, angiogenesis can serve as a way to compensate for the lack of oxygen and nutrients to the tissue. In case of a failure to adequately compensate for or repair the damage, the endothelium may suffer from loss of function, which makes the tissue susceptible to developing permanent tissue damage or pathological conditions such as coronary artery disease or diabetic ulcerations [1,2].

Endothelial progenitor cells (EPCs) are commonly used in in vitro and in vivo research. As their name suggests, EPCs are cell populations capable of differentiating into mature endothelial cells and are therefore a valuable target in regenerative medicine, especially in conditions characteristic of ischemia and in the need for vascular regeneration [19]. EPCs are usually isolated from the bone marrow, peripheral blood or umbilical cord blood [17]. EPCs are also present in fat tissue, but their laborious isolation and long culture periods make fat tissue a challenging cell source for therapeutic purposes [20]. A possible isolation purpose would be the joint isolation of EPCs and ASCs for an autologous cell culture. EPCs are commonly used in regenerative therapy research along with ASCs or their extracellular vesicles and with various mature endothelial cells, including human umbilical vein endothelial cells (HUVECs) and human adipose-derived microvascular endothelial cells (HAMECs) [21,22,23]. In addition, endothelial colony-forming cells (ECFCs), also known as outgrowth endothelial cells (OECs), are a subpopulation of EPCs that can be isolated from adult peripheral blood or umbilical cord blood [19,24]. Named after their tendency to form colonies when plated, ECFCs are widely used in research due to their high proliferation ability [24].

## 3. Adipose-Derived Mesenchymal Stem/Stromal Cells

### 3.1. Basic Characteristics of ASCs

Adipose-derived mesenchymal stem/stromal cells derive from the mesenchyme [6]. Due to their many favorable characteristics, ASCs have been under extensive research in recent years. Human ASCs (hASCs) possess a multilineage differentiation potential towards several lineages, including osteogenic, chondrogenic, adipogenic, myogenic, neurogenic, endothelial and epithelial lineages [4,25,26,27]. Human ASCs can be easily isolated from white adipose tissue—more specifically, from the stromal vascular fraction in liposuction material [6,25]. Liposuction yields an abundant number of hASCs without significant donor site morbidity in comparison with bone marrow-derived mesenchymal stem/stromal cells (BM-MSCs) [6,28]. In addition, hASCs can be maintained in vitro with a stable population doubling time with low levels of senescence, and they produce certain growth factors and cytokines, including insulin-like growth factor (IGF)-1, vascular endothelial growth factor (VEGF)-D and interleukin (IL)-8, in higher numbers than BM-MSCs do [29,30]. Moreover, hASCs can modulate the immune system, which makes them an important component in the research of inflammatory diseases [31].

Flow cytometry assays have revealed the cluster of differentiation (CD) molecules CD29, CD73, CD90 and CD105 as common positive phenotypical surface markers of hASCs [32,33]. However, some of the studies have also identified the presence of CD34, which has been termed as an unstable positive marker [32,34,35].

### 3.2. Angiogenic Properties of ASCs

**Figure 1:**
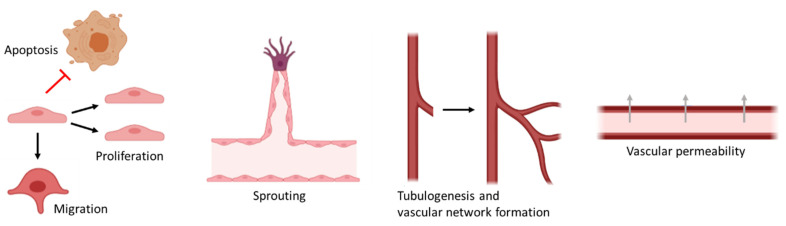
Mechanisms of angiogenesis induced by adipose-derived mesenchymal stem/stromal cells (ASCs). ASCs promote endothelial cell (EC) proliferation and migration while inhibiting apoptosis. Proliferating and migrating ECs form tubular structures, and this initial vascular network is further organized by vessel sprouting, i.e., formation of new vessels from existing ones in response to angiogenic stimuli. In mature blood vessels, ASCs regulate vascular permeability. Figure created using BioRender.

Human adipose-derived mesenchymal stem/stromal cells can promote angiogenesis (Figure 1), and they exert their pro-angiogenic effects mainly via paracrine secretion [6]. The hASC secretome is rich in several growth factors and cytokines, many of which are known to be pro-angiogenic. These include VEGF, fibroblast growth factor 2 (FGF-2, also known as basic fibroblast growth factor), platelet-derived growth factor (PDGF), transforming growth factor β (TGF-β) and hepatocyte growth factor (HGF) [8,34,36]. This abundance of growth factors makes ASCs appealing as they can affect many distinct pathways and mechanisms for angiogenesis.

VEGF is a key growth factor in endothelial sprouting, and it induces proliferation and migration of endothelial cells together with FGF-2 and PDGF [37,38,39,40,41,42]. VEGF is known to promote angiogenesis and vasculogenesis (de novo formation of blood vessels) and to regulate vascular permeability [43]. Furthermore, VEGF and FGF-2 have been shown to inhibit endothelial cell apoptosis [44,45]. FGF-2 further participates in extracellular matrix degradation, which is an important step in angiogenesis [42]. TGF-β supports the formation of tube-like structures and is an essential factor in vascular development [46,47], while HGF promotes endothelial cell growth and motility [48].

### 3.3. ASC Differentiation into Endothelial Cells

In addition to secreting paracrine factors, hASCs can support angiogenesis by differentiating into endothelial cells [4,34,35,49,50]. Table 1 illustrates different factors inducing the differentiation of ASCs towards endothelial lineage. Planat-Benard et al. [34] were among the first groups to demonstrate the endothelial potential of cells derived from the stromal vascular fraction by culturing the cells on a semisolid medium where the cells spontaneously started expressing CD31 and von Willebrand factor (vWf). Furthermore, in vivo ischemic hindlimb models have shown increased blood flow in the ischemic limb with positive staining for human CD31, showing that adipose lineage cells can incorporate into the vessel walls even with slightly varying test conditions [4,34,35].

In vitro culture conditions are a major factor influencing endothelial differentiation of hASCs. Endothelial differentiation is usually achieved by using a type of endothelial cell growth medium supplemented with growth factors and proteins, such as VEGF and IGF-1, that are known to be crucial for the differentiation process [4,35,53,54]. In addition, the endothelial cell secretome induces ASC differentiation [58]. Endothelial differentiation is detected by the presence of endothelial cell markers and biochemical processes, namely CD31, vWf, VEGF receptor 2 (VEGFR2), VEGFR1, vascular endothelial cadherin (VE-cadherin), endothelial cell nitric oxide synthase (eNOS) and acetylated low-density lipoprotein (ac-LDL) uptake [4,35,54].

It has been shown that three-dimensional (3D) culture conditions promote endothelial differentiation of hASCs by upregulating angiogenesis-promoting proteins, including VEGF, HGF, matrix metalloproteinase (MMP)-9 and IL-8 [49]. Interestingly, Park et al. [49] also found that hASCs expressed hypoxia-inducible factor-1a (HIF-1α) only in 3D cultures, not in 2D cultures. HIF-1α expression, also according to its name, is induced in cells in hypoxic conditions, which in turn induces the expression of several growth factors and proteins, including VEGF, VEGFR2, PDGF-BB, IL-8, TGF-β1, tyrosine kinase with immunoglobulin-like and epidermal growth factor-like domains (Tie-1) and nitric oxide synthase [59]. Therefore, hypoxic conditions during the cell culture can enhance the endothelial differentiation of hASCs through the upregulation of paracrine factors [57].

#### 3.3.1. Fibroblast Growth Factor 2 Regulates Endothelial Differentiation of ASCs

**Figure 2:**
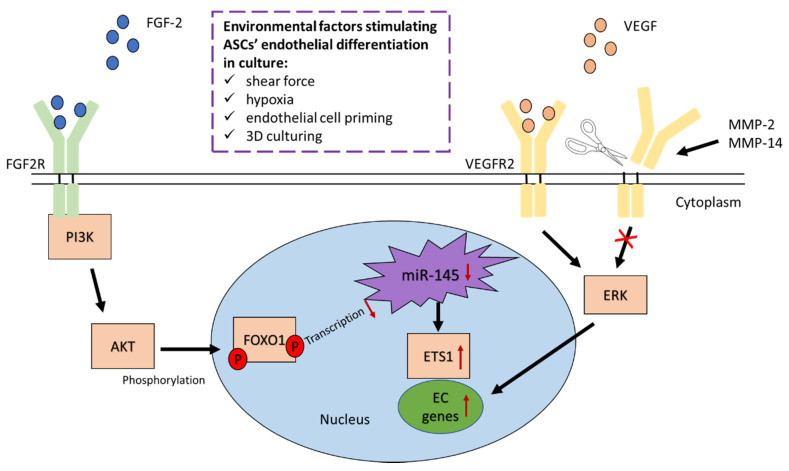
Major factors affecting adipose-derived mesenchymal stem/stromal cell (ASC) differentiation into endothelial cells (ECs). Fibroblast growth factor 2 (FGF-2) activates the phosphoinositide 3-kinase (PI3K)/protein kinase B (AKT) pathway, leading to the phosphorylation of forkhead box protein O1 (FOXO1), which decreases its transcriptional activity. As a result, the levels of miR-145 decrease and the levels of its downstream target V-ets avian erythroblastosis virus E26 oncogene homolog 1 (ETS1) increase, which upregulate the transcription of EC-related genes. VEGF, on the other hand, activates ERK signaling pathway via VEGFR2 activation, leading to upregulation of EC genes, while MMP2 and -14 induce VEGFR2 cleavage and decrease in downstream signaling. Additionally, environmental factors such as shear force, hypoxia, EC priming of ASCs and 3D culture conditions enhance endothelial differentiation of ASCs.

FGF-2 has been identified as a key component in the endothelial differentiation of ASCs. Removing FGF-2 from the culture medium of ASCs can result in reduced expression of endothelial markers such as CD31, vWf and eNOS, which indicates reduced differentiation potential [51]. Surprisingly, removing VEGF, IGF-1 or epidermal growth factor (EGF) from the culture medium did not have such an affect. In particular, the effect of removal of VEGF from the culture medium seems curious considering its close relation to angiogenic processes. Moreover, it has been observed that blocking the FGF-2 receptor in vitro results in a significantly reduced differentiation potential in hASCs and in the inability to form capillary networks, thus highlighting the importance of FGF-2 in hASC differentiation into endothelial cells [56]. A co-stimulatory effect of FGF-2 and VEGF on hASCs has been suggested, where the two growth factors strongly promoted the proliferation, migration and endothelial differentiation of hASCs in comparison with a control culture medium [56]. An in-depth study of the possible mechanism of FGF-2 revealed that activation of the FGF-2 receptor activates the protein kinase B (AKT) pathway, which leads to the suppression of forkhead box protein O1 (FOXO1) transcriptional ability (Figure 2) [60]. This in turn leads to decreased miR-145 expression, a regulator of V-ets avian erythroblastosis virus E26 oncogene homolog 1 (ETS1), which is an angiogenic transcription factor. As a result, the transcriptional activity of ETS1 is increased, thus also inducing the differentiation of hASCs into endothelial cells. These results are consistent with those of Bekhite et al. [57], who confirmed that blocking the AKT pathway abrogated the differentiation process.

#### 3.3.2. VEGFR2 and VEGFR3 Activation Induces Endothelial Differentiation of ASCs

Matrix metalloproteinase-mediated regulation of endothelial differentiation of ASCs has been suggested in recent studies. MMP-2 and MMP-14 have been shown to be upregulated during the differentiation of ASCs, which seems to have a regulatory effect on the differentiation process [50]. Silencing of MMP-2 and MMP-14 leads to increased tube formation and expression of endothelial markers. The suggested mechanism involves VEGFR2 cleavage, as it has previously been shown that MMP-2 enhances the cleavage of VEGFR2, the binding site of VEGF [61]. The presence of MMP-2 and MMP-14 led to higher VEGFR2 cleavage in comparison to a culture where MMP-2 and MMP-14 were silenced, thus resulting in lower overall expression of VEGFR2 (Figure 2) [50]. Consequently, the functionality of differentiated ASC-ECs and the expression of the angiogenesis-related proteins CD31 and VE-cadherin are decreased, suggesting that VEGFR2 activity is essential in the differentiation process. However, silencing MMP-2 or MMP-14 significantly reduces the migration capability of ASCs, a not so desirable effect for the function of ASCs [52]. Previous studies also showed that the production and secretion of MMP-2 in endothelial cells remain high when the cells still function more as individuals [62]. When the cells develop more cell–cell contacts and tubular structures, MMP-2 is downregulated.

In addition, the extracellular signal-regulated kinases (ERK) pathway has a crucial role in the differentiation of ASCs into endothelial cells through the VEGF/VEGFR pathway [52]. After 10 days of culture in endothelial cell growth medium (EGM) with silencers for MMP-2 and MMP-14, increased ERK phosphorylation was observed (Figure 2). It has previously been reported that silencing of MMP-2 and MMP-14 leads to increased VEGFR2 activity [50]. Furthermore, inhibition of the ERK pathway along with silencing of either MMP-2 or MMP-14 results in decreased expression of CD31 and VE-cadherin, which demonstrates that ERK/VEGFR2 activity is essential in the differentiation of ASCs [50,52].

Endothelial differentiation of ASCs is further stimulated in hypoxic conditions when combined with treatment of VEGF and BMP4, which increase the expression of EphrinB2 via its demethylation and induce endothelial cell markers, including CD31, VEGF-R2 and VE-cadherin, in ASC cultures [63]. The differentiated cells show improved capillary tube formation and branching and take-up of ac-LDL in vitro and enhanced angiogenesis in the ischemic hindlimb of diabetic mice in vivo. EphrinB2 is known to promote endothelial sprouting by regulating the internalization of VEGFR3, the receptor for VEGF-C, which is required for activation of its downstream signaling cascade involving the small GTPase Rac1, Akt and ERK [64]. These results suggest that ERK phosphorylation is tied to activation of both VEGFR2 and VEGFR3, which play a significant role in the differentiation process of ASCs into endothelial cells.

## 4. ASC—Endothelium Crosstalk

### 4.1. ASCs as Pericytes

Pericytes are multipotent cells that reside in the vascular basement membrane surrounding blood vessels and have a pivotal role in the maturation of vascular networks [15,65]. They can influence proliferation, maturation and sprouting of ECs and have an important role in stabilizing vessel walls by stimulating basement membrane matrix assembly [65,66].

Pericytes and ECs communicate with each other by direct cell–cell contacts and via paracrine signaling [65]. Most of the pericyte–EC interactions are mediated through the secretion of paracrine factors, which include TGF-β and angiopoietin 1 (Ang-1). Direct cell–cell interactions include exerting contractile force onto an endothelial cell, thus potentially altering its behavior [67]. Specifically, the mechanical stimulation by pericytes towards adjacent vascular endothelial cells can modulate cell–cell interactions and thereby influence angiogenesis in the microvascular niche.

**Figure 3:**
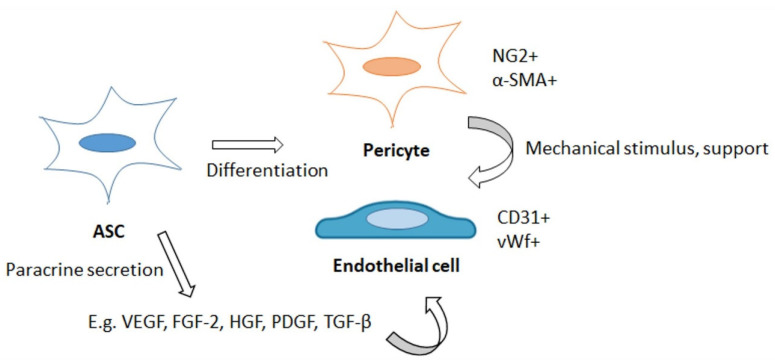
ASCs stimulate angiogenesis via paracrine secretion of growth factors, which regulate endothelial cells, and by differentiating into ECs expressing, e.g., CD31 and vWf, or by developing pericyte characteristics, including expression of NG2 and α-SMA. Pericytes provide an additional mechanical stimulus and support for ECs to stabilize developing vascular structures.

In vitro and in vivo studies performed on ASCs have shown that ASCs are often found lining the outside of microvessels, and that they take a stabilizing role by differentiating into pericytes and by expressing common pericyte surface proteins such as a late pericyte marker α-smooth muscle actin (α-SMA), neural/glial antigen 2 (NG2) or platelet-derived growth factor receptor β (PDGFRβ) [5,68,69,70]. IGF-1 upregulates α-SMA in ASCs, which indicates that it may induce ASCs’ differentiation towards pericyte-like cells [71]. Studies in mice have shown that intravitreal injection of ASCs promotes retinal microvascular stabilization suggesting that an injection of ASCs could protect microvessels from retinal vasculopathy, a disorder of retinal blood vessels [68]. Figure 3 summarizes the angiogenesis-promoting properties of ASCs.

Culture conditions affect the expression of α-SMA in hASCs. A spheroid culture induces α-SMA expression in an hASC monoculture, and α-SMA-expressing hASCs were found to surround the microvessel-like structures in an EC/hASC co-culture [8]. It is of note that in a monolayer culture, hASCs expressed only a small amount of α-SMA. In another study, neither hASCs nor ECs expressed α-SMA when cultured on tissue culture plastic [72]. These findings suggest that hASCs show a more prominent pericyte phenotype in a 3D environment compared with a standard monolayer culture.

Interestingly, when cultured on the opposing sides of an ultrathin porous membrane with HUVECs, hASCs showed dual differentiation into hASC-pericytes and hASC-ECs [73]. A subpopulation migrated across the ultrathin membrane and started expressing CD31 in an EC-like manner, while another subpopulation remained on the membrane and orientated nearly perpendicular to the hASC-ECs and showed an increased expression of NG2.

A comparison of HUVEC/hASC and HUVEC/BM-MSC co-cultures demonstrated that both co-culture systems show signs of vessel maturation by MSCs differentiating towards a perivascular cell type as indicated by positive staining for NG2 and basal lamina proteins [70]. Nitric oxide (NO) has been shown to direct the phenotype that hASCs assume in a co-culture environment with HUVECs [74]. However, in contrast to the findings of Pill et al. [68], a comparison of HUVEC/hASC and HUVEC/BM-MSC showed that a hydrogel that releases nitric oxide directs the hASCs to adapt an EC-like phenotype, whereas the BM-MSCs adapt a pericyte-like phenotype [74]. In a monoculture environment with NO, hASCs express more CD31 and VEGFR2 compared to BM-MSCs, which relates closely to EC function during angiogenesis. In contrast, BM-MSCs produced more Ang-1 than hASCs did, which plays an important role during vessel stabilization. In addition, in the co-culture environment with NO, BM-MSCs moved within close proximity of the HUVECs and expressed more α-SMA compared with the HUVEC/hASC co-culture, whereas hASCs formed an EC-like 2D layer on the hydrogel surface. Both co-cultures formed vessel-like structures, but the HUVEC/BM-MSC co-culture formed more mature networks. Moreover, an in vivo wound healing assay supported the previously listed results of BM-MSCs adapting a pericyte-like phenotype in NO gel, and accelerated wound healing was observed in comparison with the hASC group. Even though this study suggests that BM-MSCs are a superior option compared to hASCs in relation to pericyte-like behavior in co-cultures and wound healing, the difficulty of obtaining BM-MSCs in a clinical setting makes them a more complicated option.

### 4.2. Co-Cultures of ASCs and Endothelial Cells

Promoting vascularization via co-culture of a vasculogenic cell type and an adipose-derived mesenchymal stem/stromal cell type has recently attracted much attention. A 3D co-culture system of endothelial cells and ASCs is a promising approach to overcome the limitation of a single EC culture, which is unable to form stable networks in vitro when aiming to support the rehabilitation of injured tissue [8,70]. Moreover, in vitro engineered tissue constructs often result in inadequate vascularization after implantation that could be overcome with sustained growth factor secretion provided by additional ASCs. The effects of stem/stromal cell presence in an EC culture have been studied with both animal and human cells. Table 2 summarizes in vitro studies involving co-cultures of ASCs and ECs.

Many studies have indicated that the presence of stem/stromal cells in a co-culture system modulates endothelial cells and can induce angiogenesis [8,78]. Rat adipose-derived mesenchymal stem/stromal cells promote angiogenesis by inducing HUVEC tubulogenesis and expression of VEGF, Ang-1, VEGFR2 and Tie-2 in vitro [78]. Murine ASCs have shown the ability to modulate human ECFC function in vitro and to form vascular networks in vivo [86]. In addition, murine ASCs secrete pro-angiogenic factors, namely VEGF, HGF, platelet-derived growth factor 2 (PlGF-2), MMP-3, MMP-9 and members of the IGF binding proteins, which promote proliferation, migration and tube formation of ECFCs in vitro. In contrast to human ASCs, murine ASCs do not secrete FGF-1 or FGF-2 according to Lin et al. [86].

#### 4.2.1. Human ASCs Promote Tube Formation in Co-Culture Systems and Matrices

Distinct scaffold materials have been applied to support vascularization in co-cultures. In a 3D fibrin matrix, HUVECs were shown to organize into prevascular-like structures when co-cultured with hASCs [8]. The co-culture spheroids formed a branched interconnected network expressing α-SMA, CD34, HGF, MMP-1 and MMP-2. In addition, lumen formation was observed. On a polycaprolactone (PCL)/gelatin nanofiber surface, co-culture of HUVECs and ASCs exhibited increased expression of CD31, suggesting improved endothelization [83]. HUVECs co-cultured with ASCs in hyaluronic acid/gelatin gel or in a 3D-printed PCL/hydroxyapatite nanocomposite scaffold showed improved formation of capillary networks and expression of angiogenic factors [75]. Klar et al. [10] established a co-culture of human adipose-derived CD31+/CD34+ ECs and CD31-/CD34+ mesenchymal stem/stromal cells in collagen hydrogel where the co-culture condition was observed to promote capillary network formation whereas EC monoculture did not. A co-culture system in fibrin using ECFC- and hASC-coated microcarrier beads was also shown to form mature branched tubular structures, whereas a monoculture resulted in incomplete vessel-like structures [72]. In addition, MMP-14 expression was detected in endothelial cells lining the vessels, which was suggested to indicate a role for fibrin degradation. Similar results were seen without microbeads, which suggests that the use of carriers may be unnecessary. In contrast, Strassburg et al. [21] did not observe significant enhancement of prevascular-like structure formation in an indirect HUVEC/hASC co-culture separated by a membrane with a 0.4-mm pore size in Matrigel but interestingly only in an EPC/hASC co-culture. Moreover, a co-culture of hASCs and EPC spheroids in a rat collagen matrix resulted in longer sprouts, which was not the case in a HUVEC/hASC co-culture.

Co-culturing of osteodifferentiating hASCs and human microvascular dermal endothelial cells promotes angiogenesis and EC recruitment [80]. Osteodifferentiation was accomplished by culturing hASCs in an osteogenic medium with dexamethasone for seven days, which was enough to induce early differentiation but still maintain stemness. The results showed that the EC migration potential was significantly higher when co-cultured with osteodifferentiating hASCs in comparison with undifferentiated hASCs. In addition, when co-cultured in Matrigel, the capillary network formation had higher complexity with a higher count of nodes, junctions and segment lengths when compared to a co-culture with undifferentiated hASCs. In terms of enhancing vascularization in non-osteogenic lineages, the conditioned medium (CM) of osteodifferentiated hASCs could be tested in EC network formation. However, a close analysis of the possible osteogenic effects of the CM in the use of EC differentiation would have to be made. These results suggest that various matrices can promote the formation of vessel-like structures, which is more dependent on the cell type and other culture conditions. For the formation of persistent networks, it is necessary to further consider the spatial organization of the co-cultured cells.

#### 4.2.2. Direct Contact between the Cell Types Is Essential for Vascular Network Formation

It has been shown that direct contact or close proximity of hASCs to ECs is essential for mature network formation [69,72,76]. This was observed by culturing HUVEC or human peripheral blood-derived ECFC monocultures in hASC supernatants and comparing the results to a co-culture system in fibrin clots with hASC-coated beads [69]. Both EC types started forming networks in close proximity to the beads. Interestingly, seeding hASCs on top of the fibrin clots resulted in ECFCs forming a mature network faster than HUVECs and in a more stable network overall for both EC types, whereas direct seeding of hASCs together with ECs into the fibrin clot resulted in network degradation after one week of culture. A co-culture system utilizing ECFCs and hASCs has the advantage of being an autologous system, making it a potential option for clinical applications. It has also been observed that co-culture of hASCs with HUVECs over a porous membrane improves the differentiation and angiogenic potential of hASCs compared to a non-porous model [73]. The porous membrane allows partial direct contact between the two cell types. In this model, hASCs orientate perpendicularly to polarized HUVECs and express signs of pericyte-like behavior.

Noting that the lymphatic system is also an essential part of a functional tissue, Knezevic et al. [77] showed that a triculture of human dermal microvascular blood endothelial cells (BECs), human dermal microvascular lymphatic endothelial cells (LECs) and hASCs can form separate, morphologically differing endothelial and lymphatic networks in a fibrin hydrogel with a VEGF-C supplement to support LEC growth [77]. In contrast, in the absence of hASCs, neither endothelial cell type was able to form networks. As in the previously mentioned studies, it was also observed that direct cell–cell contact between endothelial cells and hASCs is required for network formation as both endothelial cell types failed to form networks in the absence of hASCs or when cultured just with hASC-conditioned media. This model needs further in vivo testing but could possibly enhance the survival of engineered grafts.

#### 4.2.3. Comparison of Different Cell Types in Co-Culture Settings

As previously mentioned, different cell types may have diverse effects on the formation of tubular structures. When comparing the co-cultures of HUVEC/hASC and HUVEC/BM-MSC, it was found that both co-cultures support the formation of vascular-like structures and induce angiogenesis in vitro and in vivo [8,76]. Ma et al. [76] showed that HUVEC/BM-MSC and HUVEC/hASC co-cultures have an equal ability to induce angiogenesis in collagen, which was not the case in monocultures, suggesting that a supporting cell type and direct contact are essential in network formation and stabilization, possibly through paracrine factors secreted by the hASCs. Similarly, Pill et al. [70] compared an HUVEC co-culture with either human BM-MSCs or hASCs in fibrin. They found that both co-culture systems remained stable over the course of 3 weeks, but the HUVEC/hASC co-culture system formed more junctions and a higher network density than the HUVEC/BM-MSC co-culture over the same time period. The results indicate that both co-culture systems are suitable for vascular tissue engineering, but considering the greater stability and higher network density in the HUVEC/hASC co-culture, hASCs seem to have a stronger angiogenic potential. As previously mentioned, Kang et al. [74] showed that BM-MSCs have a stronger tendency to differentiate towards a pericyte cell type under nitric-oxide-containing conditions. The authors suggest that the differences of those cultures should be considered when choosing a co-culture cell type [70].

A comparison between human adipose-derived microvascular endothelial cells (HAMECs) and HUVECs cultured together with either hASCs or human foreskin fibroblasts showed that HAMEC/hASC co-culture produces stable and organized vessel-like networks faster than the other cell combinations do [22]. In addition, HAMEC/hASC combination also expressed mature vessel biomarkers such as CD31 and α-SMA, while the other cell combinations did not express such vessel complexity nor such a large number of oriented vessel segments as the HAMEC/hASC group. Therefore, the HAMEC/hASC combination seems the most promising option to induce vascularization and also offers an autologous co-culture system.

#### 4.2.4. Special Characteristics of the Co-Culture Secretome

Understanding the molecular mechanisms of and secretome influence on endothelial cells and adipose stromal cells in co-culture systems is of great importance when aiming to establish a functioning co-culture system for tissue engineering applications. Some mechanisms have been unraveled, but many remain unestablished.

Despite the similarity between MSC populations derived from different tissues and with respect to cell choice for co-culture systems, it has been found that rat ASCs secrete higher amounts of VEGF and HGF than BM-MSCs [87]. Similarly, in a study comparing the co-cultures of HUVEC/hASC and HUVEC/BM-MSC, it was found that VEGF gene expression was higher in the HUVEC/hASC co-culture compared with the HUVEC/BM-MSC co-culture [70].

In a co-culture system with ASCs, additional IGF-1 exposure influenced mouse ECs via activation of the phosphoinositide 3-kinase (PI3K)/AKT signaling pathway and by enhancing the expression of angiogenesis-related growth factors [71]. In particular, an increase in the gene expression levels of VEGF, FGF-1, PDGFB, MMP-2 and MMP-9 in ECs was observed. In ASCs, additional IGF-1 resulted in the upregulation of pericyte-related genes and proteins, namely α-SMA, VEGF, MMP-2 and MMP-9. Furthermore, it has been shown that PDGFRβ (CD140b) signaling in hASCs mediates angiogenesis in human retinal endothelial cells [11]. PDGFRβ knockdown in hASCs influences their paracrine secretion profile by downregulating proteins such as IL-6 and IL-10 and by upregulating proteins such as tumor necrosis factor (TNF)-α, TNF-β and VEGF. Overall, IGF-1 is a strong inducer of the secretion of angiogenesis-related growth factors and proteins in both ASCs and ECs and can potentially be used to enhance the angiogenic process in a co-culture system. Moreover, PDGFRβ has an important role in hASC ability to assist endothelial cells in vascular network formation.

Interestingly, activin A, a member of the TGF-β superfamily, influenced the secretome of an EC/hASC co-culture where the concentrations of Ang-1, HGF and stromal cell-derived factor 1 (SDF-1), also known as C-X-C motif chemokine 12 (CXCL12), were significantly smaller than in the secretome of an hASC monoculture [9]. Surprisingly, the accumulation of VEGF increased by four-fold in the EC/hASC co-culture in the presence of activin A. Despite the increased accumulation of VEGF, the net effect of EC/hASC CM did not promote vasculogenesis, which indicates that Ang-1, HGF and SDF-1 have significant pro-angiogenic effects independent of VEGF. In hypoxic conditions, decreased levels of IL-8, monocyte chemoattractant protein (MCP)-1 and VEGF were observed in an ASC/HDMEC co-culture compared with normoxia, while secretion of IL-6 was increased [85]. This result may show the relevance of using co-cultures to treat ischemia and induce angiogenesis considering the importance of IL-6 in these processes [88,89]. Zhang et al. [79] demonstrated increased BMP-2 secretion in an ASC/HUVEC co-culture compared with ASC monoculture and enhanced VEGF secretion in the co-culture compared with HUVEC monoculture. Interestingly, secretion of these cytokines was further increased when they applied electrical stimulation for cells via culturing in an electrically conductive polypyrrole/chitosan scaffold. These effects offer potential benefits for inducing angiogenesis or bone regeneration.

#### 4.2.5. Tubulogenesis-Influencing Factors in Co-Cultures

Paracrine factors can influence tubulogenesis in a co-culture system of ECs and hASCs. For example, IGF-1 and the activation of PDGFRβ have a great influence on the vascular network formation process and hASC pericyte-like behavior. In a co-culture system, added IGF-1 was observed to enhance the length and width of vessel-like structures compared to a co-culture without additional IGF-1 [71]. On the other hand, PDGFRβ knockdown results in decreased migration capabilities of hASCs and their adhesion to extracellular matrix [11]. Consequently, when the effects of PDGFRβ are not in place, hASCs’ ability to support vascular network formation is greatly reduced.

Activin A is known to be secreted by hASCs [49,90]. In addition, it has been observed to direct the crosstalk between hASCs and ECs [9]. Human ASC CM strongly enhanced the cell survival and proliferation of human microvascular endothelial cells (HMECs) and the migration of human cord blood-derived endothelial cells (CBD-ECs) and HUVECs compared to control medium. In contrast, the levels of activin A were increased as a result of EC/hASC co-culture, and EC/hASC CM showed angiostatic activity by decreasing EC numbers by 30% and by inhibiting the migration of CBD-ECs and HUVECs. The results suggest that activin A modulates the secretome of hASCs and ECs, thus affecting vessel formation. This is the first study to suggest that direct interaction of hASCs and ECs results in an angiostatic function rather than a pro-angiogenic function.

Pre-culturing of ASCs in EGM induced the formation of cord-like structures in their co-culture with HUVECs, while similar structures were not observed in ASC/HUVEC co-cultures with no pre-culturing [82]. These results indicate that endothelial differentiation of ASCs improves tubulogenesis in a co-culture.

Introducing flow to the co-culture may affect tubulogenesis. Microfluidic devices can be used to mimic the physiological vascular microenvironment, where the tissue is perfused to provide nutrients and for waste removal. Growth factor concentration gradients created by microfluidics in an HUVEC/hASC 3D co-culture support endothelial cell sprouting and initial network formation [81]. However, direct flow through the gel removes the secretome of hASCs, which impairs mature network formation. Therefore, the use of microfluidics is only beneficial with mature lumenized vessels.

#### 4.2.6. Endothelial Cells Affect the Angiogenic Potential of hASCs

Although most of the research focuses on the ability of hASCs to promote endothelial cell function and angiogenesis, some studies also point out ways in which ECs or their secretome influence hASCs, particularly in culture conditions. Studies have shown that activin A is induced in hASCs in response to EC exposure, which inhibits vascular network formation [9]. Similarly, inhibiting activin A secretion in hASCs increased network density in a parallel test. Furthermore, hASCs’ ability to promote vascular network formation was reduced in a secondary culture after exposure to ECs in vitro. Moreover, activin A upregulated the expression of Flt-1 (VEGFR1) in ECs, which is known to be a scavenger receptor for VEGF, thus facilitating the effect of VEGF on angiogenesis.

In contrast to the study of Merfeld-Clauss et al. [9], the priming of hASCs with HUVEC secretome enhanced the proliferation, angiogenesis and wound healing abilities in a diabetic environment, created by the addition of glucose to the culture medium of both cell types, which suggests a strong paracrine effect unaffected by direct cell–cell contact [58]. In more detail, it was found that the priming significantly improved the proliferation of hASCs without additional fetal bovine serum in the HUVEC CM in comparison with a diabetic control. In addition, a significant increase in hASC mRNA concentrations of CD31, vWf and eNOS was detected, which suggests that priming may trigger endothelial differentiation. Moreover, HUVEC-primed hASCs formed capillary-like structures in Matrigel and had an improved microvessel count unlike unprimed controls, demonstrating a strong pro-angiogenic effect of hASC priming with HUVEC CM. Previously [91], the angiogenic potential of adipose-derived mesenchymal stem/stromal cells was reported to decrease in a diabetic environment, which is consistent with Fromer et al. [58]. Cianfarani et al. [91] also showed that diabetic conditions decrease hASC ability to promote angiogenesis; therefore, it can be concluded that HUVECs secrete factors that enhance hASCs’ pro-angiogenic ability in diabetic conditions [58]. An advantage of HUVEC priming in terms of clinical applicability is the fact that no additional growth medium or serum is needed. The authors suggest identifying the active factors of priming by making HUVECs deficient in a certain cytokine individually. It is probable that direct cell–cell contact is needed for the induction of activin A secretion in hASCs, which would explain why HUVEC secretome had pro-angiogenic effects instead of angiostatic effects.

### 4.3. Co-Transplantation of ASCs and Endothelial Cells In Vivo

In recent studies, the co-transplantation of ASCs and ECs in vivo has shown superior effects on the formation of vasculature over a single cell type transplantation. In a study by Wu et al. [92], ASCs and EPCs from peripheral blood alone or in combination were applied as a wound-healing treatment in mice. Co-transplantation resulted in increased blood vessel density and angiogenesis and an accelerated rate of wound closure. However, the authors used double the amount of cells in the co-transplantation versus the single cell type transplantation, which may accentuate the results. Interestingly, ASC treatment alone resulted in improved angiogenesis compared with EPCs. In radiation-induced ulcers in mice, ASC/ECFC spheroid treatment using hyaluronic acid as a vehicle improved wound healing and vascularization in the wound compared with ASC or ECFC treatment [82]. Traktuev et al. [93] transplanted human ASCs, cord blood-derived EPCs or ASCs and EPCs together subcutaneously in a collagen–fibronectin matrix into mice. The ASC–EPC implant showed improved vasculature and tighter association with tissue as well as multilayered vessels, while ASC or EPC implants showed no or only few vessels, respectively. Vessel formation was dependent on PDGF-BB signaling, and most of the ASC–EPC implant vessels were functional and filled with blood, demonstrating connections with the host vasculature when evaluated 14 days post-implantation. Kuss et al. [75] surgically implanted 3D-printed scaffolds containing ASCs or ASCs and HUVECs into mice, the latter of which resulted in improved formation of microvasculature and anastomosis with the host vasculature. In a study by Lin et al. [86], mouse ASCs were subcutaneously co-transplanted into mice with human cord blood-derived ECFCs in a Matrigel scaffold and compared with MSCs from bone marrow, skeletal muscle and myocardium. The results showed an extensive network of blood vessels where ECFCs lined the lumen and MSCs occupied the perivascular niche, while no vascularization was observed when ECFCs were transplanted alone. No differences with respect to vascularization were detected between different MSC populations.

These results agree with a study where ASCs were combined with HUVECs in collagen gels and implanted subcutaneously into mice, and the implant was compared with a BM-MSC/HUVEC implant [76]. Both implants showed equal angiogenic capacity in vivo and anastomosis with the host vasculature after 7 days. However, when Lu et al. [94] implanted ASCs subcutaneously into nude mice with ECFCs in a Matrigel scaffold, they were shown to provide improved formation of functional vessels compared with implants containing MSCs from an umbilical cord (UC-MSC) or endometrium (E-MSC) in addition to ECFCs. All co-transplantations were superior to ECFCs transplanted alone, which formed only very few vessels. Souza et al. [82] applied subcutaneous injection of EGM-2 pre-cultured ASCs together with HUVECs in type I collagen into mice and observed improved survival of HUVECs and the presence of functional blood vessels sixty days after transplantation. Lower vascular density was found after transplantation of normal ASCs with HUVECs, suggesting that endothelial priming of ASCs improves their angiogenic properties.

In order to treat ischemia, Lu et al. [95] injected either ASCs, UC-MSCs or E-MSCs with ECFCs into mice induced with hindlimb ischemia and showed that the combination of ASC–ECFC significantly promoted perfusion recovery and limb salvage compared with other MSC cell types. These studies suggest that ASCs may more effectively promote vascularization by ECs compared with some MSC populations, but this is not consistent since some studies showed equal effects with respect to different MSC origins. Moreover, it has been shown that the number of transplanted cells makes a difference with respect to blood vessel formation. Co-transplantation of 10 million ASCs with ECs resulted in a significant increase in vessel growth compared with 5 million ASCs with ECs [94].

#### The Effect of EC Origin on the Formation of Vascular Structures In Vivo

To determine whether the source of ECs is critical for the vascular assembly process, different EC populations have been tested in co-transplantation. Implants containing HUVECs, human placenta-derived ECs (Pl-ECs) or human adipose tissue ECs (AT-ECs) with ASCs transplanted into mice in a collagen–fibronectin gel all showed formation of vascular structures [93]. However, ASC/AT-EC implants showed the most prominent vascular network. In another study, EPCs from human peripheral blood or HUVECs were combined with ASCs in a fibrin matrix and implanted on the chorioallantoic membrane (CAM) of fertilized chicken eggs [94]. The results indicated that ASCs provoke the migration of blood vessels into the fibrin construct either alone or when co-delivered with EPCs or HUVECs. Significantly more capillary-like structures and more perfused blood vessels were observed in the ASC-HUVEC implant compared with the ASC-EPC implant. When EPCs or HUVECs were transplanted alone, no blood vessels or only few blood vessels were observed. Based on these results, it appears that the origin of ECs influences the vascularization potential, with mature ECs serving as a more potent alternative over EPCs or ECFCs. On the other hand, no significant formation of vascular structures occurs after transplantation of ECs alone when implanted subcutaneously using a scaffold material, while ASCs induce angiogenesis even when transplanted alone. It has been suggested that the scaffold matrix is not necessary for vasculogenesis after co-transplantation as cells delivered in media induced similar CD31-positive, functional vessels compared with cells transplanted in a collagen–fibronectin gel [93]. It is possible that ASCs provide enough support for ECs to facilitate angiogenesis. The role of ASCs as a perivascular cell type after co-transplantation has been indicated in several studies [10,86,93].

## 5. Extracellular Vesicle-Facilitated Crosstalk between ASCs and Endothelial Cells

In recent years, there has been significant increasing interest in extracellular vesicles (EVs) and their potential in angiogenesis and tissue regeneration. Extracellular vesicles play an important role in intercellular communication [96]. EVs can alter the behavior of target cells through their cargo, which consists of proteins, lipids, mRNA and microRNA. Most cell types secrete EVs, including ASCs, endothelial cells, neuronal cells, epithelial cells, cancer cells, platelets and several types of immune cells. There are several subcategories of EVs that vary in their characteristics, including exosomes and microvesicles [97]. Exosomes are released into the extracellular space by fusion of the multivesicular body with the plasma membrane, and their bilayer membrane consists of membrane proteins such as tetraspanins (CD9, CD63 and CD81) [96]. Microvesicles are formed as a result of external budding of the parent cell’s plasma membrane, and they express CD40 on their surface [96].

Not all EVs have the same qualities. Tissue source has been found to affect EV cargo [98,99]. For example, a comparison of EVs from different tissue sources regarding the effects on neuronal growth revealed that EVs from menstrual fluid MSCs had enhancing effects on neurite outgrowth compared with BM-MSC EVs, umbilical cord MSC EVs and chorion MSC EVs [99]. In addition, a comparison of hASC EVs and human BM-MSCs regarding their ability to promote tube formation and mitochondrial respiration in vitro indicated that hASC EVs outperformed BM-MSCs and that culture conditions also affected the results—more specifically, EVs from spheroid cultured hASCs had a greater effect [98]. These results suggest that special attention should be paid to the purpose of the study and the culture conditions in the selection process of MSC cell type. As the paracrine mechanisms and the secretome of MSCs differ depending on their tissue origin, MSCs should be standardized in order to consider them as a form of therapeutics [100]. Therefore, it would be important to establish concrete parameters to obtain a signature of MSCs with respect to their pro-angiogenic role and high-quality control prior to the purification of cell-secreted factors and EVs. To improve MSCs, their secretome characterization and the reproducibility of co-culture systems between donors and different cell passages, and to standardize MSCs from different sources, novel technologies such as label-free functional tests would be advantageous to profile cells and to control their quality [101].

### 5.1. Advantages of EVs

EVs have various advantages over stem cell therapy. First, stem cells may be subject to uncontrolled differentiation, become tumorigenic or undergo spontaneous transformation, whereas EVs do not directly possess this quality [102,103,104]. Concerning tumorigenicity, ASC-based cell therapy does have the risk of the stem cells promoting the growth of pre-existing tumors by promoting angiogenesis, a trait with contradicting results in ASCs where several studies speak in favor of their safety [104,105,106]. However, EVs have been found to transfer oncogenic proteins from cancer cells to cancerous cells lacking that specific protein, thereby also transferring the oncogenic activity [107]. Therefore, EVs do possess the ability to transfer potentially harmful cargo in certain instances. Second, hASCs may cause pulmonary embolism in large doses—again, a quality that EVs lack due to their small size [108]. Third, EVs as such are more stable, and therefore, they are more suited to handle storage than living cells, even though leakage from EVs may occur [109,110]. Fourth, EVs may possess the quality of homing to certain tissues depending on their source tissue, which would be a valuable characteristic for targeted drug delivery [111]. However, it is not clear how extensive this homing ability is. The disadvantage of EVs is that their cargo is static—that is, more of the desired cargo cannot be produced after administration, whereas stem cell therapy allows the administered stem cells to produce more paracrine factors and EVs [108]. It naturally follows that the therapeutic dose and efficacy of EVs must be clearly defined. Fifth, the overall poor survival rate of mesenchymal stem/stromal cells when injected or infused remains a challenge [112,113]. Therefore, due to these advantages over the use of living cells, EVs possess high potential for cellular-based therapies.

### 5.2. EVs Derived from Adipose Tissue Mesenchymal Stem/Stromal Cells

EVs from adipose-derived mesenchymal stem/stromal cells (ASC-EVs) have been found to carry a cargo capable of altering the function and phenotype of their target cells [114]. EVs from porcine adipose tissue transport gene regulatory information that can modulate angiogenesis and other cellular pathways. EVs can also have a different miRNA/mRNA/protein profile compared to their parent cell, containing a cargo that is enriched in terms of particular miRNAs/mRNAs/proteins. Eirin et al. [115] found that the miRNAs enriched in porcine ASC-EVs compared to corresponding ASCs were miR-148a, miR-532-5p, miR-378 and let-7f, but overall readings were detected from a total of 386 miRNAs. MicroRNA-148a targets transcription factors regulating angiogenesis and is known to inhibit tumor angiogenesis and reduce angiogenic sprouting of ECs [115,116,117]. The other enriched miRNAs target several transcription factors and genes related to angiogenesis and apoptosis [118,119,120]. In addition, the mRNAs enriched in porcine ASC-EVs have been found to encode transcription factors involved in angiogenesis, TGF-β signaling and Golgi apparatus proteins [115]. Another analysis of the miRNA cargo derived from hASCs confirmed two members of the let-7 family (let-7i-5p and let-7f-5p) that have been shown to take part in angiogenesis [121].

Another extensive analysis of porcine ASC-EVs revealed that EVs include miRNA and mRNAs related to transcription factor activity and thus gene expression, whereas the protein content is linked to signal transduction pathways [114]. Out of 413 identified miRNAs, miR-140-3p, miR-183, miR-222 and miR-378 were enriched in EVs in comparison with ASCs. miR-378 is known to promote MSC survival and vascularization in hypoxic conditions [122]. In addition, 255 mRNAs were enriched in EVs compared with ASCs, containing genes related to transcription in particular, while 277 proteins related to extracellular matrix, glycoproteins, angiogenesis, TGF-β signaling, inflammatory response and blood coagulation were enriched in EVs [114]. Interestingly, the analysis also showed that the mRNA cargo in EVs does not transcribe the protein cargo in EVs.

### 5.3. PDGF Enhances the Pro-Angiogenic Potential of ASC EVs

PDGF is crucial for the recruitment of endogenous pericytes by inducing their migration and proliferation during vascular development, thus favoring vessel formation [123,124]. In addition, PDGF stimulates endothelial cells and induces the transition of mesenchymal cells into vascular cells [124,125,126]. Lopatina et al. [127] have shown that PDGF increases the secretion of hASC EVs and their pro-angiogenic protein content compared to untreated EVs, creating an enhanced pro-angiogenic effect. After PDGF exposure, the EVs contained proteins such as various MMPs and thrombopoietin, and the proliferation of HMECs was enhanced. In addition, the PDGF-EV cargo contained the tyrosine kinase receptor c-KIT and its ligand stem cell factor (SCF), which were partly responsible for the enhanced effect of PDGF-EVs since the blocking of c-KIT and SCF significantly decreased the angiogenic effects.

### 5.4. The Effect of Human ASC-EVs on Endothelial Cells

Several studies have shown that ASC-EVs can promote angiogenesis in vitro and in vivo, as illustrated in Table 3. Cellular uptake assays have indicated that HUVECs and human microvascular endothelial cells (HMECs) internalize hASC EVs and that internalized EVs accumulate around the nucleus [114,127,128]. In addition, hASC EVs enhance cell migration of HUVECs and promote proliferation and vessel-like structure formation in a dose-dependent manner in human microvascular endothelial cells (HMECs) [127,128,129]. Pretreatment of hASCs with endothelial differentiation medium can upregulate the secretion of EVs [129]. Liang et al. [12] established that hASC exosomes promote angiogenesis in vitro by upregulating Ang-1 and Flk-1 (VEGFR2) in HUVECs. An in vivo study in mice showed that co-injection of HUVECs and hASC exosomes promoted vascular structure formation to a higher degree than a single treatment of HUVECs did when cells and exosomes were implanted subcutaneously within Matrigel.

A study conducted on HUVECs, human fibroblasts and keratinocytes revealed that hASC EVs promote cell proliferation and migration of all mentioned cell types [13]. In addition, hASC EVs promoted angiogenesis most likely by upregulating, among others, VEGF, VEGFR2, PDGF-A and FGF-2 in HUVECs. Moreover, activation of the AKT and ERK signaling pathways by EVs was observed in all cell types.

### 5.5. The Role of Selected ASC-Derived MicroRNAs in Endothelial Cells and Angiogenesis

**Figure 4:**
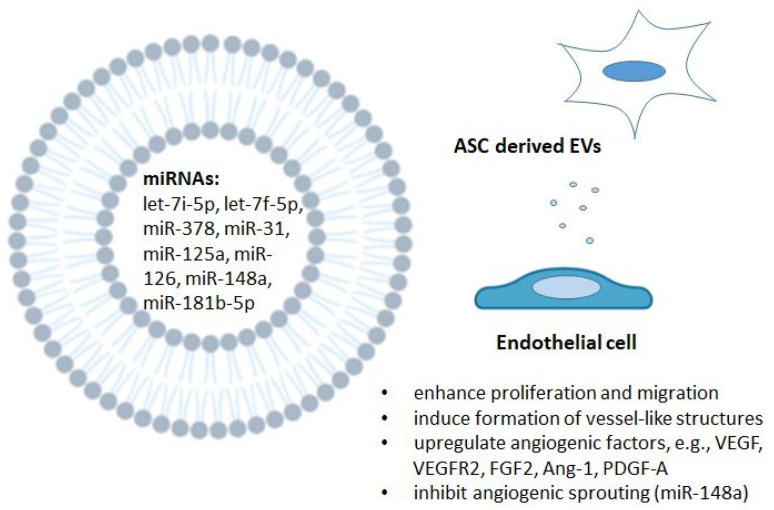
The miRNA cargo of ASC-EVs known to have effects on endothelial cells. Figure created using BioRender.

The cargo of ASC-EVs is able to modulate endothelial cell behavior (Figure 4). It has previously been established that miR-31 has a critical role in the angiogenic function of EPCs, and inhibition of the miR-31–miR-720 pathway may lead to impaired angiogenesis [144]. In addition, VEGF treatment of HUVECs and ischemic conditions have been shown to elevate miR-31 levels [145,146]. Subsequently, the microRNA has been found to be enriched in EVs, and miR-31 has been identified as a contributor to in vitro HUVEC vessel formation and migration and in vivo vessel formation in mice [129]. The angiogenesis-promoting effect was seen especially with endothelial differentiation medium-pretreated hASC EVs. Moreover, the target gene for miR-31 was found to be factor-inhibiting hypoxia-inducible factor 1 (FIH-1), which may mediate the pro-angiogenic effect of miR-31. Another study that analyzed the effects of miRNA on angiogenesis found that an enriched content of miR-125a in hASC exosomes represses expression of the angiogenic inhibitor delta-like 4 (DLL4) and promotes the formation of endothelial tip cells [12].

It has been shown that exosomes from miR-126-overexpressing murine ASCs decreased myocardial injury in an in vivo rat model by increasing angiogenesis and decreasing inflammation, apoptosis and fibrosis [23]. Similar results were seen in control ASC exosomes, but the results were enhanced in miR-126-enriched exosomes. In vitro studies indicated that miR-126-overexpressing ASC exosomes had a superior protective effect against hypoxia-induced cell injury of myocardial cells. In addition, the exosomes increased the migration and angiogenic capability of EPCs.

### 5.6. Hypoxic Conditions Augment the Pro-Angiogenic Properties of ASC-Derived EVs

Since hypoxia is present in several diseases, such as cardiovascular and metabolic disorders and cancer, and EVs are proposed to play a role in hypoxia-induced processes, it is important to evaluate the effect of hypoxia on EVs themselves. A comparison of hASC exosomes derived in hypoxic (h-exos) and normoxic (n-exos) conditions revealed that the EV uptake rate of exosomes derived under hypoxic conditions by HUVECs was significantly higher in comparison to the normoxic group [140]. In addition, h-exos have been shown to enhance the migration and tube-forming ability in HUVECs compared to that of an n-exo treatment [142]. The factors involved in the angiogenesis-promoting effects were concluded to be increased VEGF, VEGFR2, VEGFR3, EGF, FGF, MCP-2 and MCP-4 contents in h-exos and the increased activation of the PKA signaling pathway after h-exo treatment [140,142]. Another study also established enhanced tube formation by h-exos where the effect was comparable to that in positive control conditions containing an EGM-2 culture medium with additional VEGF [143].

Rat ASC-derived exosomes promote the migration and tube formation of brain microvascular endothelial cells (BMECs) after oxygen–glucose deprivation [139]. More specifically, miR-181b-5p in ASC exosomes was identified to target transient receptor potential melastatin 7 (TRPM7) and upregulate VEGF and HIF-1α in BMECs. Increased levels of the let-7 family, hypoxia-related miRNAs, in EVs from ASCs cultured in hypoxic conditions have also been found to promote the proliferation, migration and tube formation potential of vascular endothelial cells [130]. The suggested mechanism is the let-7/argonaute 1 (AGO1)/VEGF pathway where AGO1 is targeted by the let-7 family, which in turn increases the mRNA expression and translation of VEGF. Previous studies have shown that the let-7/AGO1/VEGF pathway is a key element in the de-suppression of VEGF and is essential for VEGF translation in hypoxic conditions [147]. This indicates that tube formation is strongly mediated by EVs and not only by direct growth factor and cytokine secretion.

The effects of hypoxia-derived exosomes and normoxia-derived exosomes have also been compared with respect to fat grafting in an in vivo setting. Both types of exosomes can promote the survival of a fat graft, but co-transplantation of h-exos has an enhanced ability to promote survival and neovascularization in the transplanted tissue in comparison with n-exos [141]. Both h-exos and n-exos reduce the infiltration of inflammatory cells around the grafts, enhance blood perfusion and reduce necrosis in the graft tissue [141,142]. Taken together, these results indicate that both types of exosomes can support angiogenesis, but exosomes from hypoxia-pretreated ASCs have a higher pro-angiogenic capability than those derived from normoxic ASCs.

### 5.7. Negative Impact of Obesity and Metabolic Syndrome on the Angiogenic Potential of ASC-EVs

EVs have been suggested to serve as novel prognostic biomarkers for lipid metabolism. ASC-EVs from pigs with metabolic syndrome (MetS) showed altered miRNA cargo and impaired tube formation and migration of HUVECs compared with ASC-EVs from lean pigs [148,149]. Specifically, MetS-EVs were enriched in eight miRNAs compared with 14 distinctly enriched miRNAs in lean ASC-EVs [148]. The target genes of the miRNAs enriched in MetS-EVs were involved in the development of MetS and its complications. Therefore, alterations in miRNA cargo may limit the therapeutic use of ASCs in subjects with MetS. In a similar way, obesity has been shown to decrease the angiogenic potential of EVs derived from visceral and subcutaneous hASCs by reducing their cargo of miR-126, VEGF and MMP-2 [138]. The reduced content of miR-126, VEGF and MMP-2 results in an impaired ability to support tube-like structure formation and migration in HUVECs. As it seems that the pathophysiological conditions affect the angiogenetic potential of hASC EVs, and since liposuction procedures are often performed on obese individuals, further studies are needed to ensure the clinical efficacy of hASC EVs when engineering therapeutic applications.

## 6. Physical Forces Influencing the Potential of ASCs and Their EVs for Angiogenesis

In addition to the effect of the biochemical microenvironment, the human body is subjected to mechanical forces. It is known that hemodynamic forces influence cell differentiation and vascular adaptation [55]. Therefore, the combination of chemical and mechanical factors has been studied in terms of differentiation. In a study by Colazzo et al. [55], hASCs were subjected to four different conditions of shear stress: shear stress + VEGF, shear stress-VEGF, static + VEGF and static-VEGF. The shear stress + VEGF condition resulted in changes in the hASC phenotype, such as expression of vWf, VEGFR1 and eNOS after seven days in culture, which was not detected in a VEGF-only culture. In addition, a longer culture of 14 days resulted in the expression of CD31, VEGFR2 and VE-cadherin. These findings indicate that realistic factors, such as shear forces, may be an important stimulator to take into consideration when designing cell therapy or engineered vascular grafts, and that not only soluble factors promote cell differentiation.

In terms of scaffold material development for cell transplantation, different materials have been compared for co-transplantation and combined with low-intensity pulsed ultrasound stimulation (LIPUS). Kang et al. [150] applied collagen and collagen/hyaluronan (HA) matrices to co-deliver ASCs and HUVECs subcutaneously in rats which were exposed to LIPUS. They showed that in groups exposed to LIPUS, scaffolds containing HA exhibited more blood vessels than the scaffolds without HA did. However, in groups that did not receive LIPUS treatment, no difference was observed between the materials. Overall, the LIPUS treatment augmented angiogenesis.

In addition, physical signals may affect the secretion and cargo of EVs. Magnetic fields are known to stimulate cell function and cellular processes in various ways. They can affect cell proliferation and differentiation, induce angiogenesis and osteogenesis and inhibit apoptosis [151,152,153,154]. It has been shown that a static magnetic field enhances the secretion of EVs in equine ASCs and increases the concentration of angiogenesis-inducing VEGF and osteogenesis-inducing bone morphogenic protein 2 in EVs compared to conditions without a magnetic field [155]. In a parallel study, an increase in the shedding of EVs in the presence of a static magnetic field was also reported in equine ASCs but not in canine ASCs [156]. The effects of a magnetic field seem to vary depending on the tissue origin, as a magnetic field increased the proliferation rate of equine ASCs but not canine ASCs.

## 7. Implications of ASCs and Their EVs towards Clinical Application

### 7.1. A prevascularized Transplant for Wound Treatment

In comparison to the use of a cell suspension or cell delivery within a matrix for transplantation, a prevascularized transplant could avoid the issue of cell survival in the tissue. Therefore, Klar et al. [10] generated a dermo-epidermal skin substitute that was formed by a prevascularized collagen hydrogel containing human adipose tissue-derived ASCs and ECs and covered with human keratinocytes. The transplant was applied in vivo into full-thickness wounds in immunodeficient rats. After only 3–4 days, the tissue analysis revealed functional, blood-perfused and connected capillaries formed by human and host vessels. Furthermore, after 7 days, capillaries showed lumen formation and coverage by pericytes. These results suggest that a prevascularized transplant may induce more rapid angiogenesis in tissue compared with cell delivery. The results of Klar et al. [10] and Traktuev et al. [95] further suggest that adipose tissue-derived cells themselves are a prominent option to form vascular structures and to support blood perfusion in vivo. Hence, adipose tissue could be recommended as the most suitable cell source to induce angiogenesis and to treat wounds or ischemia.

### 7.2. ASC-Derived EVs in Fat Grafting

In vivo studies in mice have revealed that EVs derived from ASCs can support the survival of fat grafts by aiding angiogenesis and increasing the volume retention rate [128,130]. Interestingly, it has also been discovered that murine ASC-derived exosomes support fat grafting in a manner comparable to that of ASCs [131]. The exosomes support fat grafting by enhancing angiogenesis and early inflammation. Co-transplantation of fat grafts and EVs resulted in a greater amount of CD31^+^ vessels and improved volume retention of the fat grafts. The results indicate that hASC EVs could potentially be used as an addition to fat grafting, but clinical studies are needed to confirm their effectiveness.

### 7.3. ASC EVs Facilitate Angiogenesis in a Diabetic Environment and during Wound Healing

A diabetic environment is known to decrease hASC function [58,91]. It has been shown that a diabetic environment results in downregulation of genes related to key cell functions in hASCs [132]. These include factors related to migration, survival, inflammatory function and angiogenesis, such as C-X-C chemokine receptor type 4 (CXCR4), CXCR7, SDF-1, chemokine (C-C motif) ligand 2 (CCL2) and angiopoietin-like 4 protein (ANGPTL4), which were significantly downregulated in hASCs from patients with diabetes (d-hASCs) in comparison with healthy donors [132,157,158,159]. Interestingly, when diabetic hASCs were transfected with healthy donor hASC EVs (nEVs), there was a significant increase in the migration ability in vitro and in vivo [132]. The treatment of d-hASCs with nEVs resulted in the downregulation of miR-29c and miR-150, the former being known to be upregulated in a diabetic environment and to induce cell apoptosis and the accumulation of extracellular matrix proteins [160]. In addition, upregulation of CXCR4, CXCR7, SDF-1, CCL2 and ANGPTL4 was observed compared to untreated d-hASCs [132]. These observations indicate a promising opportunity to alter the function of d-hASCs for their therapeutic use in diabetic patients.

Studies have shown that hASC-derived exosomes bear wound-healing abilities in a diabetic environment [133]. Human ASC exosomes may alleviate diabetic foot ulcerations by reducing inflammation and promoting angiogenesis in the tissue. Even though anti-inflammatory and pro-angiogenic effects were observed in normal hASCs, this phenomenon was most strongly expressed in exosomes derived from nuclear factor-E2-related factor 2 (Nrf2)-overexpressing hASCs, a transcription factor that plays a crucial role in fighting oxidative stress. In addition, ASC exosomes, particularly those enriched with circular RNA mmu_circ_0000250, promoted wound closure by improving microvascular development in an in vivo wound healing model in diabetic rats [135]. Exosomes derived from ASCs have already been formulated into a wound-healing gel, which was shown to support wound healing by promoting proliferation and neovascularization in an in vivo wound healing assay in a diabetic environment [134]. An exosome-containing wound-healing gel would certainly be a novel method to improve the otherwise difficult healing process of diabetic ulcerations.

In vivo wound healing studies in mice have revealed that hASC-derived EV treatment significantly enhances re-epithelialization and wound closure [13]. More specifically, EV treatment enhances cell proliferation, capillary density and the number of mature vessels in the wound, indicating that EVs could potentially aid in wound healing therapies. Moreover, studies have shown that non-diabetic EV treatment of diabetic hASCs had a reducing effect on the necrotic area of a wound comparable to non-diabetic hASCs [132]. Therefore, nEV-treated d-hASCs may present a clinically applicable way to improve the function of d-hASCs in the treatment of diabetic ulcers. If clinically applicable, the exosomes derived from hASCs could possibly be used to alleviate diabetic foot ulcerations.

### 7.4. ASC-Derived EVs and Cardioprotection

Luo et al. [23] studied the effects of rat ASC exosomes in myocardial infarction and concluded that ASC exosomes significantly decrease the area of infarction. This effect was prominent with miR-126-overexpressing exosomes, which further decreased cardiac fibrosis and inflammatory cytokine expression.

It has been found that the expression levels of CXCR7 in EPCs are lower in acute myocardial infarction (AMI) patients in comparison to healthy controls, which was found to be connected to the functionality of AMI-EPCs [121]. When AMI-EPCs were treated with exosomes from sirtuin 1 (SIRT1)-overexpressing ASCs, the levels of Nrf2 and SDF-1 significantly increased in comparison with an untreated group. Consequently, the migration and tube formation of AMI-EPCs were enhanced. Furthermore, overexpression of CXCR7 helped to restore migration and tube formation in AMI-EPCs. The positive effects of SIRT1-overexpressing exosomes were also observed in vivo, where they improved the myocardial function and reduced infarct size. Elevated expression of CXCR7 was previously shown to enhance the angiogenic potential of EPCs via enhanced Nrf2 activation [161]. The SIRT1/Nrf2 signaling pathway, on the other hand, is known to have an important role in the protection against oxidative stress, and activation of that pathway can reduce myocardial ischemic injury [162,163]. It seems evident that the function of CXCR7 can be rescued by using SIRT1.

Human ASCs and their exosomes have also been shown to provide better cardioprotection in comparison with BM-MSCs or umbilical cord mesenchymal stem/stromal cells (UC-MSCs) and their exosomes [137]. All previously mentioned cell types and their exosomes inhibited cardiomyocyte apoptosis and resulted in a smaller infarction area in vivo, with hASCs and their exosomes having the greatest effect. Similarly, when neonatal rat cardiomyocytes were cultured in hypoxic conditions and subjected to exosomes from hASCs, BM-MSCs or UC-MSCs, the exosomes reduced necrosis and promoted angiogenesis by increased secretion of VEGF, HGF and FGF-2 compared to the phosphate-buffered saline control. Again, hASC exosomes had the greatest effect compared with BM-MSC exosomes and UC-MSC exosomes.

### 7.5. Clinical Efficacy of ASCs and ASC-Derived EVs in Treatment of Ischemic Diseases

To date, no clinical studies have been reported on the co-transplantation of ASCs and ECs for ischemic diseases. However, a large number of clinical trials using ASCs for the treatment of ischemia have been completed [7,164]. Although reported to be a promising cell type for the treatment of ischemic heart disease, ischemic cerebral disease, peripheral vascular diseases and wound healing, studies have shown low survival and retention of ASCs in the ischemic microenvironment and differences in the therapeutic effect due to varying administration routes of ASCs. Several clinical trials are still ongoing, most of which aim to apply allogenic ASCs for treatment of critical limb ischemia, ischemic stroke or ischemic heart failure [165]. Regarding EVs, only one study currently recruiting patients, using mesenchymal stem cell-derived exosomes for treatment of ischemia in acute ischemic stroke aiming to improve the disability of patients, is listed in the ClinicalTrials.gov database. Therefore, the evidence for EV-based therapy is still insufficient, and further in vivo studies and clinical trials are required. Clinical translation of ASC-derived EV therapies is further limited due to the lack of standardized methodology for EV preparation and characterization as well as challenges in efficient drug loading and successful targeting into desired tissues [166].

Engineering of ASCs and/or ASC-derived EVs is a current strategy that may broaden their potential for treatment of ischemic diseases. Indeed, pharmacologically modified ASCs, e.g., with rosuvastatin or the liver X receptor (LXR) agonist T0901317, have shown increased cell survival and angiogenic properties in myocardial infarction [164,167,168]. Furthermore, genetic modification via viral gene transfer has been used to induce specific gene expression in ASCs to promote their pro-angiogenic effects [7,169]. In addition, genetic modification of cells can be used to increase the levels of therapeutic cargo of cell-derived EVs, which enhances the therapeutic effect towards ischemia [170,171]. In addition to genetic modification of the parent cells, EV performance can be enhanced via chemical modification of its surface molecules or by loading therapeutic components within EVs [172,173]. These novel strategies may provide improvements in the development of EV-based therapies, while combined cell co-transplantation can also be a promising way of treating ischemic diseases in the future.

## 8. Conclusions

As endothelial dysfunction is the common denominator of numerous pathological conditions, including diabetic foot ulcers and coronary heart disease, angiogenesis and restoration of vascular structure and function are key elements in re-establishing proper organ function. Regenerative medicine aims to prevent further tissue damage due to hypoxia and to promote reperfusion. Adipose-derived mesenchymal stem/stromal cells have been under extensive research in the past two decades due to their ability to modulate the immune system and to participate in tissue regeneration. As they can easily be harvested from the adipose tissue, low donor site morbidity makes them a more favorable option over bone-marrow-derived mesenchymal stem cells. Basic research in co-cultures with endothelial cells has demonstrated the pro-angiogenic effects of ASCs through paracrine secretion or via direct cell contact where ASCs support the formation of tube-like structures. Additional IGF-1 in the culture medium promotes the expression of growth factors in ECs and ASCs that are important for angiogenesis via the PI3K/AKT signaling pathway. In addition, activation of PDGFRβ further promotes vascular network formation in vitro, whereas activin A, secreted by ASCs, inhibits vascular network formation. ASCs can differentiate into endothelial cells, especially in three-dimensional culture conditions. FGF-2 and activation of the PI3K/AKT signaling pathway are of great importance in the endothelial differentiation of ASCs. Moreover, ASCs can participate in microvessel stabilization by differentiating into pericytes.

ASC-derived extracellular vesicles have emerged as a novel possibility for therapeutic angiogenesis. In comparison to ASC-based therapy, EVs possess several advantages, including stability, small size, homing ability and lack of uncontrolled cell differentiation. According to the data obtained to date, hASC-derived EVs display high pro-angiogenic potential—a valuable quality in tissue regeneration research. Currently, it is known that ASC EV cargo contains several pro-angiogenic miRNAs, mRNAs and proteins. However, there is no consensus regarding the cargo of ASC-derived EVs, which is most likely affected by varying culture conditions and the cell origin, highlighting the importance of quality control prior to the production of ASCs and their derivative products for medical use. In addition, many questions still remain unanswered. Despite the promising in vitro and in vivo effects of extracellular vesicles, extensive clinical studies need to be conducted in order to establish a realistic picture of the applicability of EVs in therapeutic angiogenesis.

## Figures and Tables

**Table 1:** Factors inducing ASC differentiation towards endothelial cells.

Factor	Source of ASC	Authors	Details
Semisolid methylcellulose medium	Human	[34]	Expression of CD31 and vWf and formation of vessel-like structures in vitro. In vivo neovascularization. Dedifferentiation of mature adipocytes into ECs.
Endothelial cell growth medium containing VEGF and IGF	Human	[35]	Expression of CD31 and vWf in vitro. Increased capillary density and blood flow in ischemic hindlimb in vivo.
Endothelial cell growth medium containing VEGF and FGF-2, Matrigel coating	Human	[4]	Ac-LDL uptake in vitro. Expression of CD31, VE-cadherin and eNOS in vitro and in vivo. Improved blood perfusion in vivo. Blockade of PI3K inhibits differentiation.
Endothelial cell growth medium containing FGF-2	Rat	[51]	Ac-LDL uptake; expression of CD31, vWf and eNOS; and formation of tube-like structures on Matrigel in vitro. Blockade of FGF-2 inhibits differentiation.
Endothelial cell growth medium containing VEGF	Pig	[52]	Endothelial cell morphology and increase in ERK phosphorylation in vitro. ERK inhibition decreases the expression of CD31 and VE-cadherin. Blockade of VEGFR2 inhibits ERK.
Endothelial cell growth medium containing VEGF, FGF-2, EGF and IGF-1	Human	[53]	Change in morphology; induced expression of CD31, vWf and eNOS; and formation of cord-like structures on Matrigel in vitro. Improved fat graft retention and neovascularization in vivo.
Endothelial cell growth supplement, shear force	Human	[54]	Ac-LDL uptake and expression of CD31 in vitro. No expression of eNOS or vWf.
Shear stress + VEGF	Human	[55]	Expression of CD31, VE-cadherin, vWf, eNOS and VEGFR1 and -2 in vitro.
Added FGF-2 or FGF-2 + VEGF	Human	[56]	Expression of CD31, vWf, eNOS and VE-cadherin and formation of capillary-like structures on Matrigel in vitro. Blockade of FGF receptor inhibits differentiation.
Hypoxia in combination with leptin and VEGF	Human	[57]	Expression of CD31, VE-cadherin, vWf, VEGR2 and eNOS and increased sprout formation on Matrigel in vitro. Blockade of AKT inhibits differentiation.
HUVEC priming	Human	[58]	Change in morphology and increased expression of CD31, vWf and eNOS. Formation of capillary-like tube networks on Matrigel.
3D cell culturing	Human	[49]	Formation of 3D cell mass induced hypoxia and expression of VEGF, IL-8 and CD31 among other angiogenic factors. Formation of vascular structures in vivo when implanted in mice.
Silencing of MMP-2 and MMP-14	Pig	[50]	Increased expression of CD31 and VE-cadherin, formation of capillary tubes and ac-LDL uptake in vitro. Decreased cleavage of VEGFR2.

**Table 2:** Summary of in vitro studies involving co-culture of ASCs and endothelial cells to promote vascularization.

EC Type	Source of ASC	Authors	Details and Effect on Vessel Formation
HUVEC	Human	[8]	Culturing of fibrin-embedded spheroids induced organization into prevascular-like structures expressing CD34 and α-SMA.
OEC	Human	[72]	Induced formation of CD31-positive branching vessel structures in a fibrin matrix. Expression of MMP-14 in the invading sprouts. Elevated VEGF secretion.
HUVEC	Human	[75]	Improved capillary network formation and expression of CD31, vWf, VEGF and MMPs in HA/gelatin gel. Enhanced vascularization in a 3D-printed composite scaffold.
AT-EC	Human	[10]	Vascular network with continuous endothelial lumen formation.
HUVEC	Human	[76]	Induced formation of vessel-like structures on Thermanox (2D) and in collagen gel (3D).
BEC + LEC	Human	[77]	In a triculture in fibrin gel, LEC and BEC form separate networks, which are dependent on ASC contact. Lymphatic network is dependent on VEGF-C.
HUVEC, rat LMEC	Rat	[78]	Improved tubulogenesis in Matrigel. Upregulation of VEGF, Ang-2, VEGFR2 and Tie-2 in HUVECs.
EPC, HUVEC	Human	[21]	Increased VEGF secretion and formation of capillary-like structures with longer sprouts in ASC/EPC co-culture but not in ASC/HUVEC co-culture. Blockade of VEGFR2 inhibits capillary-like structure formation.
HUVEC	Human	[79]	Enhanced calcium deposition and secretion of BMP-2 and VEGF, which were further increased by electrical stimulation.
CBD-EC	Human	[9]	The co-culture induces activin A expression in ASCs and secretes lower levels of angiogenic factors compared with ASC culture.
HMEC	Human	[80]	Improved capillary network by osteodifferentiating ASCs. ECs enhance the production of VEGF, PDGF-B and FGF-2 in osteodifferentiating ASCs.
HUVEC, OEC	Human	[69]	Proximity of ASCs required for mature network formation in fibrin gel. ASCs induce and stabilize EC networks by developing pericyte characteristics and by protein secretion.
HUVEC	Human	[70]	Induced network formation and deposition of basal lamina components in a co-culture in fibrin. ASCs differentiate toward a pericyte phenotype.
HUVEC	Human	[73]	ASCs show pericyte-like behavior and differentiation into ECs in a co-culture over a porous membrane.
HUVEC	Human	[74]	ASCs exhibit EC-like phenotype in a co-culture in nitric-oxide-releasing gel. Increased sprouting in the beginning of cultures.
HAMEC, HUVEC	Human	[22]	HAMEC/ASC co-culture induces the most organized and complex vascular network expressing CD31 and α-SMA in a 3D scaffold.
Mouse BMEC	Mouse	[71]	IGF-1 enhances the formation of vessel-like structures and upregulates the expression of angiogenic factors via PI3K/AKT pathway in collagen gel.
HUVEC	Human	[81]	Indirect flow enhances EC sprouting but fails to form vascular networks in fibrin gel, while direct flow inhibits prevascular network formation.
HUVEC	Human	[82]	Pre-culture of ASCs in EGM-2 improves the formation of tube-like structures in a co-culture.
HUVEC	Rat	[83]	Enhanced CD31 expression on co-spun nanofiber substrate.
ECFC	Human	[84]	Co-culture in a hyaluronic acid gel reverses late-passage ASC senescence and shows increased amount of CD31-positive cells.
HDMEC	Human	[85]	Myofibroblast differentiation of ASCs attenuated in co-culture. Hypoxia increases expression of IL-6. Increased expression of VEGF compared with EC culture.

**Table 3:** Summary of angiogenesis-related studies involving adipose-derived mesenchymal stem/stromal cell-derived extracellular vesicles.

Context	Source of ASC	Authors	Effect of ASC EVs
Angiogenesis	Human	[127]	Stimulate in vitro and in vivo angiogenesis. PDGF enhances EV secretion in ASCs.
Angiogenesis	Human	[129]	Promote angiogenesis in vitro and in vivo via miRNA-31.
Angiogenesis	Human	[12]	Promote angiogenesis in vitro and in vivo via miRNA-125a.
Angiogenesis, fat grafting	Human	[128]	Promote angiogenesis and fat grafting in vivo.
Angiogenesis, fat grafting	Human	[130]	Improve survival of fat graft by angiogenesis promotion via let-7/argonaute 1/VEGF pathway.
Angiogenesis, fat grafting	Mouse	[131]	ASC-EVs comparable to ASCs in aiding fat graft survival via angiogenesis promotion and fat graft volume retention.
Wound healing	Human	[13]	Promote in vivo wound healing via activation of the AKT and ERK pathways. Promote angiogenesis.
Wound healing, diabetic environment	Human	[132]	Healthy EVs can upregulate the expression of genes important to wound healing. Enhance the mobility of diabetic ASCs to the wound site in vitro and in vivo.
Wound healing, diabetic environment	Human	[133]	Promote wound healing in a diabetic foot ulcer model in vivo. Enhanced effect with nuclear factor-E2-related factor 2 (Nrf2)
Wound healing, diabetic environment	Mouse	[134]	Exosome containing wound-healing gel promotes wound healing and angiogenesis in diabetic environment in vivo.
Wound healing, diabetic environment	Human	[135]	mmu_circ_0000250-modified ASC-EVs promote wound healing in vivo.
Myocardial infarction	Rat	[23]	miRNA-126 overexpression prevents myocardial damage and promotes angiogenesis in vivo.
Myocardial infarction	Mouse	[136]	SIRT-overexpressing ASC-EVs promote survival and myocardial function by promoting angiogenesis via Nrf2 in vivo.
Myocardial infarction	Human	[137]	Inhibit cardiomyocyte apoptosis, reduce infarction area and increase microvascular density in vivo.
Obesity	Human	[138]	Obesity decreases pro-angiogenic effect of EVs via impairment of miR-126 content.
Hypoxia, angiogenesis	Rat	[139]	Promote angiogenesis via miRNA-181b in oxygen–glucose deprivation in vitro.
Hypoxia, angiogenesis	Human	[140]	Hypoxia treatment of ASCs promotes EV-induced angiogenesis via protein kinase A (PKA) signaling pathway.
Hypoxia, angiogenesis, fat grafting	Human	[141]	Promote survival of fat graft by promoting angiogenesis and reducing inflammation. Hypoxia pretreatment of ASCs can enhance the effects.
Hypoxia, angiogenesis, fat grafting	Human	[142]	Hypoxia treatment of ASCs promotes EV-induced angiogenesis and fat grafting in vivo.
Hypoxia, angiogenesis	Human	[143]	EVs from hypoxia-conditioned ASCs are a more potent angiogenesis inducer than EVs without preconditioning.

## Abbreviations

3D	Three-dimensional
α-SMA	α-smooth muscle actin
ac-LDL	Acetylated low-density lipoprotein
AGO-1	Argonaute 1
AKT	Protein kinase B
AMI	Acute myocardial infarction
Ang-1	Angiopoietin 1
ANGPTL4	Angiopoietin-like 4 protein
ASC	Adipose-derived stem/stromal cell
AT-EC	Adipose tissue-derived endothelial cell
BEC	Blood vascular endothelial cell
BMEC	Brain microvascular endothelial cell
BM-MSC	Bone marrow-derived mesenchymal stem/stromal cell
CAM	Chorioallantoic membrane
CBD-EC	Cord blood-derived endothelial cell
CCL	Chemokine (C-C motif) ligand
CD	Cluster of differentiation
CM	Conditioned medium
CXCL	C-X-C motif chemokine
CXCR	C-X-C chemokine receptor
d-hASC	Diabetic human adipose-derived stem/stromal cell
DLL4	Delta-like 4
EC	Endothelial cell
ECFC	Endothelial colony-forming cell
EGF	Epidermal growth factor
EGM	Endothelial cell growth medium
E-MSC	Endometrium-derived mesenchymal stem/stromal cell
eNOS	Endothelial cell nitric oxide synthase
EPC	Endothelial progenitor cell
ERK	Extracellular signal-regulated kinase
ETS1	V-ets avian erythroblastosis virus E26 oncogene homolog 1
EV	Extracellular vesicle
FGF	Fibroblast growth factor
FIH-1	Factor inhibiting hypoxia inducible factor 1
FOXO1	Forkhead box protein O1
HAMEC	Human adipose-derived microvascular endothelial cell
hASC	Human adipose-derived stem/stromal cell
HDMEC	Human dermal microvascular endothelial cell
h-exo	Hypoxic exosome
HGF	Hepatocyte growth factor
HIF-1α	Hypoxia-inducible factor-1-alpha
HMEC	Human microvascular endothelial cell
Hsp	Heat shock protein
HUVEC	Human umbilical vein endothelial cell
IGF	Insulin-like growth factor
IL	Interleukin
LEC	Lymphatic endothelial cell
LIPUS	Low-intensity pulsed ultrasound stimulation
LMEC	Lung microvascular endothelial cell
MCP	Monocyte chemoattractant protein
MetS	Metabolic syndrome
miR, miRNA	Micro-RNA
MMP	Matrix metalloproteinase
n-EV	Healthy donor extracellular vesicle
n-exo	Normoxic exosome
NG2	Neural/glial antigen 2
NO	Nitric oxide
Nrf2	Nuclear factor-E2-related factor 2
OEC	Outgrowth endothelial cell
PCL	Polycaprolactone
PDGF	Platelet-derived growth factor
PDGFR	Platelet-derived growth factor receptor
PI3K	Phosphoinositide 3-kinase
PKA	Protein kinase A
Pl-EC	Placenta-derived endothelial cell
PlGF	Platelet-derived growth factor
SCF	Stem cell factor
SDF	Stromal cell-derived factor
SIRT1	Sirtuin 1
TF	Transcription factor
TGF-β	Transforming growth factor beta
Tie	Tyrosine kinase with immunoglobulin-like and epidermal growth factor-like domains
TNF	Tumor necrosis factor
TRPM7	Transient receptor potential melastatin 7
UC-MSC	Umbilical cord-derived mesenchymal stem/stromal cell
VE-cadherin	Vascular endothelial cadherin
VEGF	Vascular endothelial growth factor
VEGFR	Vascular endothelial growth factor receptor
vWf	von Willebrand factor
